# Widening the concept of oncogene

**DOI:** 10.18632/aging.101111

**Published:** 2016-10-31

**Authors:** Sergio Casas-Tintó, Marta Portela, Alberto Ferrús

**Affiliations:** Institut Curie, 75248 Paris, France

**Keywords:** oncogenes, metabolism, cell mechanics

As genomic cancer data bases and experimental studies progress, some genes reveal unsuspected roles in oncogenesis. We provide here a short survey on some key examples to illustrate the need to reconsider our current definition of oncogene. As age advances, somatic mutations in metabolic, signaling or regulation of apoptosis may trigger tumorigenesis, in particular if affecting damaged or precancerous cells.

Decreasing mitochondrial respiration enhances glycoly-sis triggering the “Warburg effect”. The Isocitrate dehydrogenase (IDH1) converts isocitrate to 2-oxoglutarate. Somatic mutations in cytoplasmic IDH1 and mitochondrial IDH2 are common early drivers in glioma and acute myeloid leukaemia (AML) [1], being most frequent in diffuse gliomas and secondary glioblastoma [2]. In a similar context, mitochondrial respiratory chain complexes (RC) catalyze a cascade of oxidation reactions to generate ATP. Mutations in RC complexes I and II are frequent in cancer cells, where they contribute to form reactive oxygen species which result fatal for the cell. In addition, mutations in RC components prevent the apoptosis of tumoral cells and represent bad prognosis markers in cancer [3].

Kinases activation can be the primary event during oncogenesis or a secondary event as recipient of oncogenic signaling. Tyrosine kinases (EGFR, PDGFR or Src) and serine-threonine kinases (Raf or Akt) are well characterized and lead to cell proliferation and inhibition of apoptosis. However, among the family of kinases directly involved in cancer, some are not yet considered canonical oncogenes. For example, the apoptosis signal-regulating kinase 1 (ASK1), a MAP kinase-kinase-kinase implicated in apoptosis, inflammation, proliferation and migration, activates c-Jun N-terminal kinase (JNK) and p38 and is clearly related to tumorigenesis in liver, skin, melanoma and colon cancers [4].

While these metabolic and signaling proteins could easily be accepted as new members of the oncogene class, others are unsuspected arrivals. During recent years some muscular proteins have been related to cell proliferation and oncogenic transformation. The myocyte enhancer transcription factor 2 (Mef2) synergizes with Notch to stimulate proliferation and metastasis [5]. The Troponin-Tropomyosin complex regulates muscle contraction through the acto-myosin interaction, however, we have recently described that Troponin I (TnI) is frequently mutated in human cancer and cooperates with other oncogenes to increaser cell proliferation [6]. TnI overexpression enhances PI3K pathway activity and it is necessary for tumorous growth.

SPARC (secreted protein acidic and rich in cysteine) is a conserved multifunctional extracellular matrix glycoprotein. Its expression is altered in cancer, but its effects on tumor growth are still poorly understood because SPARC seems to restrict, as well as to promote, tumor growth and metastasis. Cell competition is a process that maintains tissue health and delays aging by culling suboptimal cells. Drosophila SPARC is expressed in epithelial loser cells during competition protecting them from apoptosis. This, allows damaged cells to recover before being eliminated by their neighbors [7]. Presumably, SPARC could act as a tumor suppressor, tumor promoter or pro-invasive factor. For example: overexpression of SPARC modulates de equilibrium between cell growth and apoptosis in neuroectodermal and medulloblastoma cells. SPARC suppresses glioma proliferation in vitro and delays brain tumor growth in vivo. SPARC overexpression increases AKT phosphorylation, which is important for the antiapoptotic role, and it interacts with procaspase-8 to stimulate apoptosis which results in the potentiation of chemotherapy sensitivity in colorectal cancers [8].

Thus, it may seem appropriate to revise our current definition of oncogene in order to accommodate a growing number of gene functions that clearly synergize other classical oncogenes and that manifest as frequent mutations and bad prognosis markers in many types of tumors. In this way, an extended number of potential targets for cancer therapy will become available.
